# Molecular Mechanisms behind Inherited Neurodegeneration of the Optic Nerve

**DOI:** 10.3390/biom11040496

**Published:** 2021-03-25

**Authors:** Alessandra Maresca, Valerio Carelli

**Affiliations:** 1 IRCCS Istituto delle Scienze Neurologiche di Bologna, Programma di Neurogenetica, 40139 Bologna, Italy; valerio.carelli@unibo.it; 2 Department of Biomedical and Neuromotor Sciences, University of Bologna, 40139 Bologna, Italy

**Keywords:** optic atrophy, retinal ganglion cells, complex I, mitochondrial fusion, mitochondrial fission, mitochondria-associated membranes, calcium handling, phospholipids, mitochondrial DNA

## Abstract

Inherited neurodegeneration of the optic nerve is a paradigm in neurology, as many forms of isolated or syndromic optic atrophy are encountered in clinical practice. The retinal ganglion cells originate the axons that form the optic nerve. They are particularly vulnerable to mitochondrial dysfunction, as they present a peculiar cellular architecture, with axons that are not myelinated for a long intra-retinal segment, thus, very energy dependent. The genetic landscape of causative mutations and genes greatly enlarged in the last decade, pointing to common pathways. These mostly imply mitochondrial dysfunction, which leads to a similar outcome in terms of neurodegeneration. We here critically review these pathways, which include (1) complex I-related oxidative phosphorylation (OXPHOS) dysfunction, (2) mitochondrial dynamics, and (3) endoplasmic reticulum-mitochondrial inter-organellar crosstalk. These major pathogenic mechanisms are in turn interconnected and represent the target for therapeutic strategies. Thus, their deep understanding is the basis to set and test new effective therapies, an urgent unmet need for these patients. New tools are now available to capture all interlinked mechanistic intricacies for the pathogenesis of optic nerve neurodegeneration, casting hope for innovative therapies to be rapidly transferred into the clinic and effectively cure inherited optic neuropathies.

## 1. Introduction

**Figure 1:**
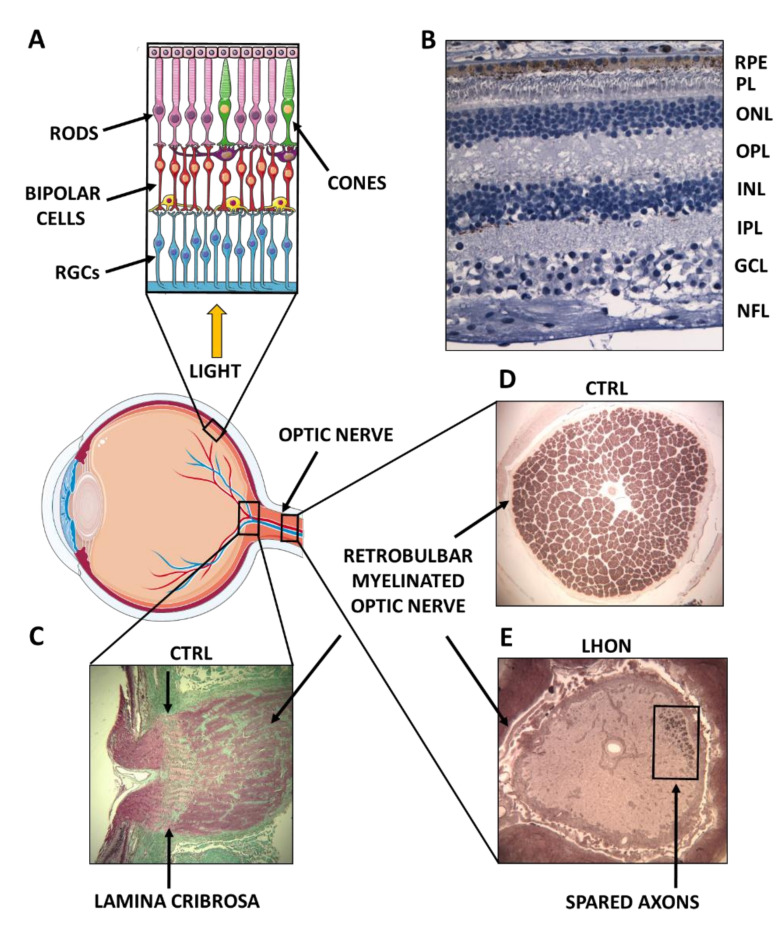
Anatomy of the human retina and optic nerve. (**A**) Graphical illustration of the eye, with the retina and the optic nerve highlighted. The three key retinal cell types implicated in the visual processing are indicated: photoreceptors (rods and cones), bipolar cells, and ganglion cells (RGCs). The pigment epithelium juxtaposed to photoreceptors, horizontal (purple) and amacrine (yellow) cells are also shown. (**B**) Histological cross-section of a normal human retina. The layers constituting the retinal tissue are indicated: retinal pigment epithelium (RPE), photoreceptors layer (PL), outer nuclear layer (ONL), outer plexiform layer (OPL), inner nuclear layer (INL), inner plexiform layer (IPL), ganglion cell layer (GCL), and nerve fiber layer (NFL). (**C**) Histological sagittal section of a normal optic nerve head (post-mortem). The lamina cribrosa, where unmyelinated axonal bundles are organized, is indicated, as well as the post-laminar retrobulbar myelinated optic nerve. (**D**) Histological cross-section of a normal optic nerve (post-mortem) is shown with myelinated bundles hosting about 1.2 million axons. (**E**) Histological cross-section of an optic nerve (post-mortem) from a patient severely affected by Leber Hereditary Optic Neuropathy (LHON), showing only a few spared axons. Images are a courtesy of Alfredo A. Sadun and Fred N. Ross-Cisneros from Doheny Eye Institute, UCLA, California, USA.

Optic atrophy may be an isolated manifestation of a mono-symptomatic disorder, but may also be a quite common feature of complex syndromes characterized by degeneration of multiple neuronal networks [1,2]. The axons forming the optic nerve originate from retinal ganglion cells (RGCs) (Figure 1), the terminal neurons sending the information elaborated by the retina to the brain cortex, through the intermediate stop, along the visual pathway, in the *nucleus geniculatus lateralis* (LGN) [3]. In addition to optic nerve damage secondary to compressive/infiltrative, inflammatory/autoimmune, or traumatic causes, the vast majority of optic nerve diseases are inherited traits, and within the inherited forms the vast majority is due to mitochondrial dysfunction [3,4].

Mitochondrial diseases are the most common genetic rare disorders, even if individual syndromes may be exremely rare [5,6,7]. Inheritance follows any of the Mendelian traits, dominant, recessive or X-linked, as over 1000 genes of the nuclear genome (nDNA) encode mitochondrial proteins, precisely 1136 according to the last version of MitoCarta 3.0 [8], including a few in the X-chromosome. Amongst the many forms, Dominant Optic Atrophy Kjer’s type (DOA) is the most frequent, and over 70% of cases can be ascribed to various types of mutations affecting the *Optic Atrophy 1* (*OPA1)* gene, encoding a key protein of the mitochondrial dynamics machinery [3,4,9]. However, of great interest is also the multicopy circular mitochondrial DNA (mtDNA), which is exclusively maternally inherited, and follows its own genetic rules resembling those of population genetics [5,6,7,10]. The most frequent disease due to mtDNA missense mutations affecting complex I, and leading to oxidative phosphorylation (OXPHOS) deficiency, is Leber’s hereditary optic neuropathy (LHON) [3,4,10]. As DOA and LHON are largely mono-symptomatic disorders, it must be remarked that an incredibly wide spectrum of other mitochondrial disorders including optic atrophy as defining feature, starting from the DOA “*plus*” [2,11] and LHON “*plus*” [2,3,4] variants, are described and continue to be described. The last in time is the spectrum associated with dominant and recessive mutations affecting the mitochondrial single stranded binding protein (SSBP1) [12,13,14]. Thus, within the frame of mitochondrial functions, a crossroad of many metabolic pathways, we have at least two major pathogenic mechanisms, possibly interconnected, which may lead to RGCs and their axons’ neurodegeneration: complex I-related OXPHOS dysfunction and mitochondrial dynamics [1,2,3,4,15,16].

The most relevant forms of isolated or syndromic optic atrophy that apparently do not fit directly the mitochondrial paradigm are those related to Wolframin, with either dominant or recessive mutations, the latter typically causing Wolfram syndrome [17]. This protein is now established to be involved with the contact points between mitochondria and endoplasmic reticulum (ER) [18,19], currently defined as mitochondria-associated ER membranes or MAMs [20]. This qualifies a third major mechanistic area for optic nerve neurodegeneration, involving the complex inter-organellar crosstalk, which, in turn, affects mitochondrial dynamics, in particular fission [21], but also quality control, specifically autophagy [22], ultimately impacting cell survival or death [20].

It must be remarked that none of these mechanistic pathways for optic nerve neurodegeneration is isolated from each other, having plenty of interconnected links, ultimately leading to a few key outcomes, such as impaired axonal transport, dendritic maintenance, myelination turnover, and neuronal cells fate [23].

In this review, we explore the three major areas outlined, as well as some newer emerging mechanisms, connecting the underlying genetic causes with the currently known mechanistic insights relevant for optic nerve neurodegeneration.

## 2. Complex I Dysfunction: The Paradigm of Leber’s Hereditary Optic Neuropathy (LHON)

LHON is the paradigm for non-syndromic optic neuropathy, and has a number of peculiar features that make it remarkable. In addition to being maternally inherited, as due to usually homoplasmic mtDNA mutations affecting complex I subunits (m.3460G > A/*MT-ND1*, m.11778G > A/*MT-ND4*, and m.14484T > C/*MT-ND6*) (Table 1), LHON is characterized by (1) male prevalence, (2) incomplete penetrance in both genders, (3) very cell-specific target, the RGCs, and, finally, (4) a subacute onset and rapidly evolving natural history, which is an unusual feature for neurodegeneration. In a limited subset of cases, we are now aware that an LHON-like disease may occur as a recessive trait, yet involving complex I components encoded by nDNA [24,25].

Thus, complex I dysfunction seems to be one primary mechanism leading to RGCs’ neurodegeneration, as at least three downstream consequences are always implicated: (1) only a partially inefficient bioenergetics, as there are multiple alternative pathways to keep mitochondrial respiration ongoing downstream complex I; (2) an increased production of reactive oxygen species (ROS), which, however, remains mostly indirectly documented; and (3) an increased propensity to apoptosis, not necessarily through the canonical pathways implying cytochrome *c*-mediated caspase activation.

Complex I, the largest of the respiratory complexes composed by about 47 subunits, catalyzes the transfer of two electrons from NADH to ubiquinone, coupled to the translocation of four protons across the inner mitochondrial membrane, to generate the proton-motive force used to ultimately synthesize ATP [26]. As far it concerns the three common mtDNA mutations associated with LHON, now that the complex I structure is much better elucidated [27], there are different types of dysfunction that may be envisaged [3]. Since the early investigations on the biochemical defect induced by LHON mutations, it was clear that only the m.3460G > A/*MT-ND1* mutation significantly reduces the enzymatic activity of complex I, as traditionally assessed by a spectrophotometer, providing donor (NADH) and acceptor (ubiquinone) substrates to run the redox reaction [28,29]. The ND1 subunit is now well defined for its role as major component of the pocket hosting the ubiquinone acceptor site [30], thus fully justifying an impaired electron flow and transfer to ubiquinone through the intermediate state of semi-quinone in the presence of the m.3460G > A/*MT-ND1* mutation [27]. In contrast, neither for the most frequent and severe m.11778G > A/*MT-ND4* mutation, nor for the milder m.14484T > C/*MT-ND6* mutation, there was a clearly documented reduction of complex I redox activity [28,29,31,32,33,34]. This finding puzzled for a while the investigators on the pathogenicity of these mutations in LHON [35]. However, complex I-driven synthesis of ATP was clearly impaired with all three LHON primary mutations, certifying their pathogenic role [36]. Interestingly, the early observation on the m.11778G > A/*MT-ND4* and m.3460G > A/*MT-ND1* mutations documenting the change in rotenone sensitivity in LHON patients [29,32] is now substantiated by the identification of binding sites to this potent complex I inhibitor in both the ND4 and ND1 subunits [37]. This confirms a structural impact of LHON mutations on complex I, possibly reflected in the coupling mechanism [37]. Based on the new understanding of the complex I structure and function, we confirm that the m.3460G > A/*MT-ND1* mutation primarily impairs electron transfer to ubiquinone, whereas we may envisage that the m.11778G > A/*MT-ND4* and m.14484T > C/*MT-ND6* mutations possibly affect proton pumping or coupling of electron transfer with proton pumping [26,27,37]. The overall understanding holds true that a combination of bioenergetics impairment and increased ROS production, still needing detailed characterization, leads to increased propensity of cells to undergo apoptosis [38,39,40].

In addition, LHON has two features that remain peculiar, the gender prevalence, as males are much more prone to undergo the disease than females, and the incomplete penetrance, as many of the mutation carriers remain lifelong unaffected [1,2,41]. Both features imply that complex I function may be compensated spontaneously to the point that the disease does not occur. Similarly, complex I function might also be modulated by external factors such as environmental exposures, which may trigger the catastrophic onset of optic nerve degeneration. The mtDNA background, as defined by the genetic variability of haplogroups, has been consistently implicated in LHON penetrance, in particular for the two mutations not affecting complex I redox activity, m.11778G > A/*MT-ND4* and m.14484T > C/*MT-ND6* mutations [42,43]. Haplogroup J, in fact, has been unequivocally associated with these two mutations [44], implying that the clustering of missense variants affecting complex I and III subunit genes in this mtDNA background may affect the overall complex I and super-complex I + III stability [45]. Interestingly, haplogroup J has also been implicated as the most sensitive to the interaction with environmental toxicants [46] that have been associated with LHON [47], including rotenone [48]. As far it concerns gender, studies based on the cybrid cell model clearly indicated that estrogens ameliorate the metabolic impairment due to LHON mutations by activating mitochondrial biogenesis, providing some insights on female protection [49]. This observation prompted the further substantiation that mitochondrial biogenesis is a compensatory strategy activated by LHON mutations in both genders, possibly by ROS signaling. Thus, the efficiency in activating mitochondrial biogenesis drives LHON penetrance, as individuals remaining unaffected are, on average, much more compensating [50]. A further byproduct of this mechanism is the well-established role played by environmental factors as disease triggers, in particular tobacco smoking [51,52], which was shown to hamper the compensatory activation of mitochondrial biogenesis in vivo and in vitro [53].

The recent identification of recessive LHON, involving mutations in the *Dna J Homolog Subfamily C Member 30* (*DNAJC30*) gene, opened a further scenario, which needs better understanding on how might be linked to the “canonical” mtDNA-related LHON [25]. In particular, this recent discovery revealed, by tracking protein turnover in patient-derived cells and a *DNAJC30*-knockout cell model, a reduced turnover of specific complex I N-module subunits, which results into complex I impairment. Thus, DNAJC30 operates as a chaperone protein needed for the efficient replacement of complex I subunits exposed to ROS and integral to a mitochondrial complex I repair mechanism [25]. Remarkably, *DNAJC30* recessive mutations express phenotypically as LHON, most probably converging on a pathological mechanism common to that of the mtDNA mutations. How this pathological mechanism translates into a catastrophic propagation of RGCs death and axonal degeneration is the next question. The current hypothesis is that the biochemical impairment of complex I reflects on key factors implicated in the peculiar neuronal architecture of RGCs. In fact, these neurons are notorious for having a long stretch of their axons unmyelinated and very energy-dependent, but also related to myelin maintenance, mitochondrial dynamics and transport along the axons, and the vascular co-participation in retinal and axonal physiology [23].

Complex I impairment emerges as a converging theme for optic nerve atrophy also from rarer forms of inherited optic neuropathies, involving genes for which the function is still incompletely elucidated. In particular, the rare recessive form of optic neuropathy associated with mutations in the *Transmembrane Protein 126A* (*TMEM126A*) gene (*OPA7*), sometimes leading to additional neurological features (auditory neuropathy, sensorimotor axonal neuropathy, mild hypertrophic cardiomyopathy), was documented to lead to partial deficiency of complex I in one patient [54]. Concordantly, two recent studies identified TMEM126A as a factor necessary for the correct complex I assembly [55,56]. Furthermore, another recessive form of isolated or syndromic optic neuropathy associated with mutations in the *Reticulon 4 Interacting Protein 1* (*RTN4IP1*) gene (*OPA10*) has been reported to present with a combined defect of complex I and IV [57], but also with a profound complex I defect when associated with the severe phenotype [58]. Interestingly, the *RTN4IP1* gene encodes a mitochondrial protein associated with the outer mitochondrial membrane predicted to interact with the partner Reticulon 4 (RTN4) localized to the ER, implicating the MAM, a further mechanism that is discussed separately.

## 3. Mitochondrial Dynamics Failure: From Dominant Optic Atrophy (DOA) to Complex Syndromes

Mitochondrial “dynamics” refers to alternative cycles of fusion, in which mitochondria merge to renew their composition and stimulate OXPHOS, and fission, in which mitochondria divide into distinct organelles, allowing mtDNA segregation or elimination of damaged/old mitochondria through a selective form of autophagy (mitophagy). In addition to their physiological role, these processes also occur in response to stress conditions or during cell death [59].

Here, we describe the basic mechanisms of mitochondrial dynamics in mammalian cells and consider the pathogenic mechanisms leading to optic atrophy due to mutations in genes encoding for the protein machinery involved in mitochondrial fusion and fission (Table 2).

### 3.1. Mitochondrial Fusion Dysfunction as the Cause of DOA or “Plus” Syndromic Forms

**Figure 2:**
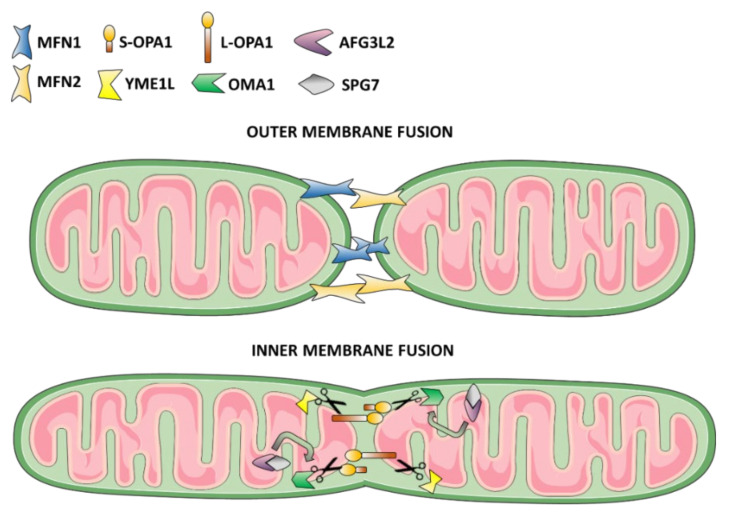
Proteins regulating mitochondrial fusion and associated with optic atrophy. A graphical representation of outer and inner membrane fusion with emphasis on proteins involved in the degeneration of optic nerve (see also Table 2).

The main pathogenic mechanism underlying DOA is definitely an unbalance of mitochondrial fusion and fission, which may be directly affected, as in the case of mutations in *OPA1* or other regulators of the dynamics machinery, or indirectly compromised, for example due to genetic defects of mitochondrial proteases involved in the OPA1 processing (Figure 2). Indeed, besides LHON, in most cases the degeneration of RGCs leading to optic atrophy is imputable to OPA1 dysfunctions. Moreover, *OPA1* is the most frequent gene associated with isolated forms of DOA [9].

The major players of mitochondrial dynamics belong to the Dynamin Related Proteins (DRP) family, consisting of proteins able to remodel organelle membrane shape through GTP hydrolysis [60]. To date, in mammals, the only factor directly regulating fusion of the inner mitochondrial membrane (IMM) is OPA1, which co-operates with others DRPs in the outer mitochondrial membrane (OMM) (i.e., mitofusins) to produce a highly interconnected and tubular mitochondrial network [59]. Thanks to a mitochondrial targeting sequence (MTS) at the N-terminal, OPA1 is addressed to mitochondria, where it is embedded into the IMM through a transmembrane domain, exposing the GTPase domain in the intermembrane space (IMS) [61]. However, in addition to this unprocessed IMM-anchored form of OPA1 (long-OPA1, L-OPA1), also a soluble form of OPA1 (short-OPA1, S-OPA1) is present in the IMS, due to the proteolytic activity of two proteases located in the IMM: the YME1-Like Protein 1 (YME1L) and the Overlapping Activity With M-AAA Protease (OMA1) [62,63,64].

The presence of both L-OPA1 and S-OPA1 in a fine-tuned balance, together with the pro-fusion lipid cardiolipin, is required for the execution of the IMM fusion [65,66,67,68]. On the contrary, an increase of OMA1 activity due to stress conditions related to OXPHOS dysfunction leads to an imbalance towards S-OPA1 forms, with consequent fragmentation of the mitochondrial network [63,69,70]. Interestingly, OMA1 activity is regulated by different mechanisms, including autocatalysis [70,71], or the degradation mediated by YME1L [72,73]. In addition, OMA1 activity is stimulated in the presence of defects in the mitochondrial protein synthesis quality control [74,75], in which a major role is played by the complex composed of AFG3 Like AAA ATPase 2 (AFG3L2) and paraplegin (encoded by *SPG7* gene) proteases. Concordantly, AFG3L2 is also part, together with YME1L, of the IMM scaffolding protein Stomatin-Like Protein 2 (SLP2), and the Presenilin-Associated Rhomboid Like (PARL) protease, of a multi-protein complex called SPY, which negatively regulates OMA1 activity, thus stabilizing L-OPA1 and inducing the so-called “stress-induced mitochondrial hyperfusion” as a survival stress response [76,77]. The composition of L- and S-OPA1 within mitochondria is also dependent on the alternative splicing of OPA1 mRNA, producing eight different transcripts that are ubiquitous, but differentially expressed in cells and tissues [78]. In fact, while the cleavage site S1 (exon 5), specific for OMA1, is present in all OPA1 isoforms, the S2 (exon 5b) YME1L specific site is present exclusively in variants 4, 6, 7, and 8. Notably, a recent study has identified an additional cleavage site called S3 (exon 4b) specific for YME1L and proposed a pro-fusion role for S-OPA1 forms derived by this processing [68]. This means that, based on the combination of OPA1 isoforms expression, there will be a variable content in mitochondria of L- and S-OPA1 [69].

The presence of multiple isoforms may be also the explanation for the multiplicity of OPA1 functions in the mitochondrial homeostasis, such as the control of cristae morphology, OXPHOS function, and mtDNA maintenance. Remarkably, while for the IMM fusion isoforms generating both L- and S-OPA1 are required, the other functions may be performed indistinctly by L- or S-OPA1 [66,79].

In the majority of cases, *OPA1* dominant mutations cause exclusively optic atrophy as clinical manifestation. However, about 20% of mutations, prominently missense mutations located in the GTPase domain, lead to syndromic phenotypes called DOA-plus that may include additional features such as deafness, peripheral neuropathy, myopathy with chronic progressive external ophthalmoplegia (CPEO), ataxia, Parkinsonism, and dementia. A few cases of biallelic *OPA1* mutations, mostly compound heterozygous, have been reported to causing a severe disease known as Behr syndrome [9]. The pathogenic mechanism of *OPA1* mutations has been extensively investigated in patients-derived cells or in animal models. It principally implicates the mitochondrial network fragmentation, cristae disorganization, OXPHOS deficiency [11,80,81,82,83,84,85,86,87,88], and mtDNA maintenance defects leading to multiple deletions in post-mitotic tissues [11,44,88,89]. The main pathological features, such as mitochondrial network fragmentation and reduced energetic capacity, have been also reproduced in induced pluripotent stem cell (iPSC)-derived dopaminergic neurons from patients carrying OPA1 mutations associated with Parkinsonism and DOA [90,91]. In this model, neuronal axons exhibited a severe depletion in the number of mitochondria, which showed also reduced motility, especially in the anterograde direction towards the synaptic terminals [92]. Moreover, enhanced autophagy and mitophagy have been documented in fibroblasts from patients carrying *OPA1* mutations [88,93,94], as well as in RGCs isolated from *Opa1* mutant mouse models [86,95,96,97]. Recently, Zaninello and colleagues demonstrated that, due to AMPK-dependent hyper-activation of mitophagy at axonal hillock, RGCs axons from Opa1 mutant mouse become depleted of mitochondria, resulting into dying back axonopathy, which ultimately hampers RGCs functioning and survival [97]. As a consequence of OPA1 deficiency, also the metabolomics and lipidomics profiles are altered in either DOA or DOA plus patients, as well as in cellular and mouse models of DOA. Alterations of purine metabolism (xanthine, hypoxanthine, and inosine), lipid metabolism (1-oleyl-rac-glycerol, rac-glycerol-1-myristate and glycerate, phosphocholine, and choline), and of few aminoacids (cystine, aspartate, glutamic acid, and urocanate) have been described in plasma from patients carrying OPA1 mutations [98]. Concordantly, variations in several phospholipids concentration have been reported in mouse embryonic fibroblasts (MEF) *Opa1*^-/-^ [99], MEF *Opa1*^-/-^ expressing different OPA1 mutants [100], and in the *Opa1*^delTTAG/+^ mouse model, specifically in the optic nerve and in plasma [101]. Thus, while alterations in purine metabolism and reduced glutamic acid are recurrent in mitochondrial diseases or in the presence of ATP depletion [102,103,104,105], changes in phospholipids, confirmed in three different models, may be the effect of mitochondrial membrane remodeling due to OPA1 dysfunction, in particular the defective mitochondrial fusion.

In the last years, thanks to next generation sequencing (NGS), novel genes have been found associated with optic atrophy, many of which modulating OPA1 activity, i.e., AFG3L2 and YME1L. Mutations in AFG3L2 and SPG7 have been initially identified as the cause of spinocerebellar ataxia type 28 (SCA28) and spastic ataxia type 5 [106,107], and hereditary spastic paraplegia type- 7 (HSP7) [108], respectively. More recently, mutations in both these genes have been reported in patients affected by DOA or syndromic diseases with optic atrophy, also including Parkinsonism [109,110,111,112,113]. As described above, dysfunctional AFG3L2-paraplegin complex triggers a stress response orchestrated by OMA1 activation [74,75], which, in turn, enhances OPA1 processing leading to an imbalance towards the soluble forms of OPA1 and mitochondrial fragmentation. Accordingly, this has been confirmed in fibroblasts derived from patients carrying *AFG3L2* mutations in the ATPase domain and affected by DOA [109,111,112]. Interestingly, patients carrying biallelic *SPG7* mutations showed hyperfused mitochondria, in contrast to what observed in *AFG3L2* mutant fibroblasts [114].

Concerning the two proteases of OPA1, while mutations in OMA1 have not been associated with human disease yet, homozygous mutations in YME1L have been reported in four patients from a consanguineous Saudi Arabian family presenting an infantile-onset mitochondriopathy with optic atrophy [115]. This mutation impaired the maturation of YME1L, leading to degradation of the mutant protein, and consequently to the proteolysis of its targets PRELI Domain Containing 1 (PRELID1), a lipid transfer protein for phosphatidic acid in the IMS, and OPA1. Fibroblasts derived from these patients showed a fragmented mitochondrial network and reduced cell proliferation. These results are coherent with what observed in the mouse model [116,117] and MEF [64,72], where loss of Yme1l accelerates the processing of L-OPA1 by OMA1 with consequent mitochondrial fragmentation. Since YME1L is also involved in the phospholipid signaling dependent on the phosphatidic acid phosphatase LIPIN1 and the Mechanistic Target of Rapamycin 1 (mTORC1) [118], alterations in the lipidomics profile of *YME1L* mutant patients may also occur, similarly to what observed for *OPA1* mutations; however, this aspect awaits to be evaluated.

The execution of mitochondrial fusion, as mentioned above, requires the coordination of two distinct processes, the outer and inner membranes fusion. The DRPs in the outer membrane ensuring this process are Mitofusins (i.e., MFN1 and MFN2), large homologous proteins with highly similar structures: the GTPase domain, transmembrane domains that are inserted in the OMM, and two heptad repeat domains (HR1 and HR2) exposed in the cytosol that allow homotypic and heterotypic interactions between MFNs located on different mitochondria. The tethering of juxtaposing mitochondria trough *trans* interactions mediated by HR2 domains represents the first step of OMM fusion. Then, the GTP hydrolysis promotes MFNs conformational change mediating mitochondria-mitochondria anchoring and fusion [59]. Although both MFN1 and MFN2 are fusion competent proteins [119,120], MFN2 is less efficient in tethering mitochondria, possibly due to lower GTPase activity compared to MFN1 [121]. Interestingly, MFN2 is also enriched at MAMs, the ER–mitochondria interface essential for efficient mitochondrial Ca^2+^ uptake and lipids transfer between the two organelles [122,123,124,125].

Mutations in *MFN2* cause the axonal form of Charcot–Marie–Tooth neuropathy (CMT2A) [126], either with dominant or recessive transmission and with the co-occurrence in some cases of optic atrophy [9,127,128]. The molecular pathogenesis of *MFN2* mutations in CMT2A remains unclear, since contradictory results have been reported. Altered mitochondrial distribution and bioenergetics defects have been described in mice overexpressing in neurons a pathogenic mutation in *MFN2* associated with CMT2A [129]. In mice overexpressing mutant MFN2 in dorsal root ganglia, mitochondrial transport, distribution, and morphology within the axons were significantly compromised, however, bioenergetics defects were absent [130,131]. While Mfn2-deficient MEF stably expressing mutant MFN2 exhibited slight mitochondrial fragmentation and increased susceptibility to oxidative stress [132], patient-derived fibroblasts carrying heterozygous *MFN2* mutations showed no alterations of mitochondrial network, together with absence of defective mitochondrial morphology and abundance, or mtDNA depletion [133]. On the contrary, fibroblasts from patients carrying biallelic *MFN2* mutations showed increased mitochondrial fusion [134]. Cytochrome c oxidase (COX) deficiency and mtDNA depletion has been observed in both fibroblasts and skeletal muscle from patients with heterozygous mutations [135]. CMT2A iPSC-derived motor neurons exhibited decreased mitochondrial content, perinuclear positioning of mitochondria and enhanced mitophagy [136]. Instead, defective ER-mitochondria contacts has been coherently reported in patients’ fibroblasts, in MEF overexpressing mutant MFN2, in mouse primary neurons and, *in vivo*, in a mouse model of CMT2A [137,138]. As a consequence of the MAM alterations, ER stress, calcium handling, and phospholipids abnormalities have been described in these models. Thus, it turns out that CMT2A may be caused by defective MAMs-correlated pathways rather than defective fusion, possibly due to the compensatory effect of MFN1 activity.

Although optic atrophy has never been reported, mutations in the *Misato Homolog 1* (*MSTO1*) gene, encoding for a pro-fusion protein, are causative for syndromic diseases characterized by muscular dystrophy, cerebellar atrophy, and pigmentary retinopathy [139,140,141,142]. Patient-derived fibroblasts showed mitochondrial network fragmentation and, interestingly, mtDNA depletion with nucleoids alterations [139,141].

### 3.2. Mitochondrial Fission Dysfunction as the Cause of DOA and Syndromic Forms

In analogy to fusion, mitochondrial fission is a process consisting of multiple sequential steps, but leading to the opposite result of scission of one mitochondrion into two daughter mitochondria.

A crucial event for mitochondrial fission is the recruitment of the cytosolic GTPase Dynamin-related protein 1 (DRP1) on the OMM and its oligomerization to form a constriction ring [59]. DRP1 is a highly conserved protein presenting four domains: the N-terminal GTPase domain, the middle domain, the variable domain, and the GTPase effector domain in C-terminal [143]. The absence of phospholipids-binding domains in DRP1 structure implies that its localization on the OMM requires adaptors proteins: the mitochondrial fission factor (MFF), the mitochondrial dynamics proteins of 49 kDa (MID49) and 51 kDa (MID51), and the Mitochondrial Fission 1 Protein (FIS1) [144], although a direct role for the latter as DRP1-receptor in mammals is still ambiguous [60]. DRP1 localizes on the OMM in specific areas, where ER and actin had previously wrapped the membrane generating pre-constriction sites [145,146]. This process allows the narrowing of the mitochondrial diameter and the formation of a DRP1-oligomeric ring-like structure surrounding the mitochondrion and enhancing the membrane constriction through GTP hydrolysis [147]. Remarkably, the ER-mitochondria contacts, which will determine the future scission sites, co-localize with replicating mtDNA nucleoids, thus ensuring the segregation of mtDNA in the newly formed mitochondria [21,148]. The ER-bound inverted-formin 2 (INF2) and the mitochondrial anchored formin-binding Spire1C cooperate to promote actin assembly at the ER-mitochondria contact sites, required for mitochondrial constriction [146,149,150]. Other proteins regulating the actin cytoskeleton are involved in this mechanism, including Myosin IIA, cofilin, cortactin, and Septin 2 [151,152,153,154]. Although DRP1 is essential for the first phases of mitochondrial fission, it may be not able to complete the scission of the mitochondrion [155], suggesting the involvement of other proteins. Recently, it has been demonstrated that Dynamin 2 (DNM2) acts downstream of DRP1 and it may be responsible for the final step of division [156]. Direct actors of the IMM constriction during mitochondrial fission have not been identified yet. However, a major role may be played by Ca^2+^ influx from ER to mitochondria at fission sites leading to IMM constriction before DRP1 recruitment on the OMM, as demonstrated by two recent studies [157,158]. Interestingly, fission of IMM may be promoted by increased levels of S-OPA1, as generated by a calcium-dependent leak in mitochondrial membrane potential and consequent OMA1 activation [157].

**Figure 3:**
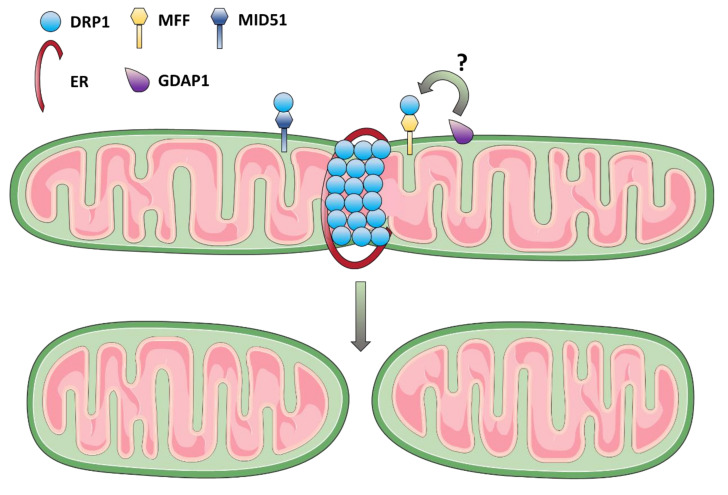
Proteins regulating mitochondrial fission and associated with optic atrophy. A graphical representation of the fission process, where the proteins involved in the degeneration of optic nerve are shown (see also Table 2).

Dysfunction of mitochondrial fission, as much as mitochondrial fusion, has deleterious effects on neuronal cell homeostasis, being indeed associated with optic atrophy and neurodegeneration (Figure 3).

In 2007, a case of lethal encephalopathy, which included optic atrophy in the clinical features, has been reported as caused by a de novo heterozygous dominant-negative mutation in *DRP1.* Patient fibroblasts showed hyperfusion of both peroxisomes and mitochondria, with depletion of peroxisomes and a few mitochondria displaying increased size and localization in the perinuclear region [159]. This phenotype closely resembled that observed in cells silenced for DRP1 or overexpressing a dominant-negative DRP1 mutant [155,160,161,162,163]. Additional cases of encephalopathy with variable clinical phenotypes caused by *DRP1* mutations, frequently de novo, have been reported thereafter [164,165,166,167,168,169], including two families with autosomal recessive inheritance [170,171]. All studies of patient fibroblasts showed, hyperfused and giant/balloon-like mitochondria, together with peroxisomal abnormalities. In one study, autophagy has also been investigated, revealing a defective mitochondrial turnover due to mitochondrial fission dysfunction [169]. Besides these complex and severe disorders, dominant *DRP1* mutations are also the cause of isolated optic atrophy undistinguishable from OPA1-DOA, as described by Gerber and colleagues. However, in these cases no alterations in the peroxisomal network were observed [172].

Similarly, mutations in the *MFF* gene have also been associated with autosomal recessive encephalopathy, again characterized by the presence of optic atrophy, and abnormal elongation of both mitochondrial and peroxisomes with diffuse cytoplasmic localization of DRP1 in patient fibroblasts [173,174]. In addition to MFF, another DRP1 adaptor, MID51 (*MIEF1* gene), has been recently associated with a form of late-onset optic atrophy. MID51 mutant proteins expressed in HeLa cells decreased the mitochondrial fusion events and fragmented the mitochondrial network [175].

Ganglioside Induced Differentiation Associated Protein 1 (GDAP1), a glutathione S-transferase enzyme that seems to be a regulator of mitochondrial fission, although with still unclear mechanism of action [176,177], is associated with a form of CMT occasionally presenting also optic atrophy [178,179]. Interestingly, overexpression of GDAP1 induces alterations of mitochondria distribution within the cell with a marked perinuclear localization [180], as already observed for DRP1 knockdown [155,160,162,163] or MFN2-mutant [136]. Concordantly, GDAP1 is an OMM protein enriched in MAMs, where it interacts with two proteins involved in mitochondrial trafficking, the Ras-Related Protein Rab-6B (RAB6B) and caytaxin [181]. A recent study demonstrated that GDAP1 deficiency disrupts mitochondrial axonal transport in both anterograde and retrograde traffic and also affects mitochondrial bioenergetics and Ca^2+^ homeostasis [182], confirming previous evidence [181,183].

Other factors involved in the mitochondrial fission process have been linked to human diseases, such as MID49, associated with mitochondrial myopathy [184], DNM2, associated with centronuclear myopathy [185], Charcot–Marie–Tooth neuropathy type 2M with neutropenia and early-onset cataract [186,187] and lethal congenital contracture syndrome [188], and INF2, associated with focal segmental glomerulosclerosis [189] and Charcot–Marie–Tooth disease with glomerulopathy [190]. Thus far, optic neuropathy has not been reported in association with these genes.

## 4. Mitochondria-Associated Membranes: The Case of Wolfram Syndrome and More

MAMs are dynamic structures regulating several intracellular pathways, such as calcium homeostasis, lipid synthesis and inter-organelles trafficking, mitochondrial dynamics, autophagy, and apoptosis [125]. These contacts are transitory juxtapositions of ER and mitochondria, separated by a distance of 10–30 nm [191], occurring without membrane fusion. It is plausible that these structures are generated through the action of specific proteins forming a bridge and tethering the two organelles [192]. In yeast, the physical connection between ER and mitochondria seems to be mediated by the ER–mitochondria encounter structure (ERMES) complex, encompassing two OMM proteins (Mdm10 and Mdm34), one protein located in the ER (Mmm1), and one in the cytosol (Mdm12) [193]. Although an ERMES equivalent complex has not been identified in mammalian cells, MFN1 and MFN2 heterodimers may mediate the tethering of ER and OMM at MAM sites [122]. Other proteins enriched in MAMs have been proposed for carrying out this function, such as DRP1 [145], the mitochondrial ubiquitin ligase, MITOL [194], the voltage-dependent anion channel, VDAC1 [195], and the phosphofurin acidic cluster protein 2, PACS-2 [196]. However, further investigations are needed to elucidate this aspect in mammals.

### 4.1. MAMs Function in Calcium and Lipids Homeostasis, Autophagy, and Apoptosis

The association of ER and mitochondria allows the exchange of molecules that are fundamental for several cellular processes. Intracellular Ca^2+^ is stored within the ER, which, under specific conditions, is released and captured by mitochondria. This process takes place at the MAM interface, and is mainly mediated by the inositol tri-phosphate receptors (IP3Rs) [197,198]. Moreover, different chaperone proteins of the ER can bind Ca^2+^, controlling its storage within ER and its flow upon stimulation of IP3Rs. For example, after ligand stimulation, IP3Rs directly interact with VDAC1 in the OMM through the chaperone glucose-regulated protein 75 (GRP75) [195,199]. Similarly, the SIGMA-1 receptor forms a Ca^2+^ sensitive complex with the chaperone glucose-regulated protein 78 (GRP78/BiP), which regulates the degradation of IP3Rs upon Ca^2+^ depletion in the ER [200]. Interestingly, the same chaperones are upregulated in the unfolded protein response (UPR), following ER stress [201]. Once released from ER, Ca^2+^ can be taken up by mitochondria passing the OMM through VDAC1 and then imported in the matrix through the IMM mitochondrial calcium uniporter (MCU) [202,203]. The mitochondrial calcium influx and OXPHOS are extremely coordinated processes. The principal driving force for the mitochondrial calcium influx is the negative membrane potential in the matrix generated by the respiratory chain. At the same time, intramitochondrial calcium boosts ATP production by OXPHOS, necessary for the functioning of calcium pumps on the plasma membrane (PMCA) and on the Sarco/endoplasmic reticulum (SERCA) [204]. Moreover, the activity of three enzymes belonging to the Krebs cycle, isocitrate dehydrogenase (ICDH), oxoglutarate dehydrogenase (OGDH), and pyruvate dehydrogenase (PDH), is regulated by mitochondrial calcium, which boosts the production of NADH to sustain OXPHOS [205,206]. In physiological conditions, the uptake of calcium is transitory and its efflux is regulated by the calcium antiporter channels [207]. However, in pathological conditions, mitochondrial calcium overload triggers an increase in ROS production, which, in turn, determines the opening of the mitochondrial permeability transition pore (mPTP), loss of membrane potential, release of cytochrome *c*, and ultimately leads to cell death [204]. Interestingly, mitochondrial calcium also regulates the secretion of several hormones from endocrine cells, such as insulin from pancreatic β-cells [208,209].

Other molecules transferred from ER to mitochondria, and vice versa through MAMs, are phospholipids, major components of the mitochondrial membranes and essential for the cristae architecture, thus impinging on the assembly of respiratory complexes in the IMM, and for mitochondrial morphology. MAMs are enriched in proteins deputed to lipid transfer and in enzymes implicated in lipid synthesis. For example, cardiolipin and phosphatidylethanolamine (PE), both crucial for mitochondrial membranes fusion, are synthesized in mitochondria starting from a major precursor deriving from ER, phosphatidic acid (PA). Through two different pathways, PA gives rise to PS and phosphatidylinositol (PI) on the ER side, whereas a series of reactions in the IMM transform PA in cardiolipin, which remains within mitochondria, primarily in the IMM. On the contrary, PS is imported in mitochondria and converted in PE, which, in turn, is transferred to the ER to generate PC, again re-imported within the mitochondria [210,211]. Protein complexes at the MAM sites mediate the inter-organelles travel of PA, PS, and PC. For example, in mammalian cells the complexes PRELID1/TP53 Regulated Inhibitor Of Apoptosis 1 (TRIAP1) and PRELID3b/TRIAP1 may mediate the transfer of PA and PS, respectively [212,213]. Recently, it has been demonstrated that Mfn2 binds and transfers phosphatidylserine (PS) across mitochondria-ER contact sites [214]. STARD7, steroidogenic acute regulatory protein (StAR)-related lipid transfer (START) protein, mediates the trafficking of phosphatidylcholine to mitochondria [215,216] and intramitochondria [217].

Remarkably, when exposed in the OMM, cardiolipin participates in the execution of programmed cell death interacting with cytochrome *c*, Bid and caspase-8 [218]. Similarly, the PRELID1/TRIAP1 complex exerts anti-apoptotic function through the control of CL synthesis in the IMM [212]. Moreover, MAMs contains a few enzymes regulating the ceramides metabolism, suggesting that a pool of these pro-apoptotic lipids resides at the ER-mitochondria contacts [219]. Thus, MAMs are implicated in the cell death program, not only through the regulation of calcium fluxes, but also through lipid synthesis and trafficking.

Lastly, MAMs might provide lipid and membranes for the autophagosome formation during autophagy [220]. The process of autophagosome formation initiates from PI synthase-enriched sites in the ER, thus requiring PI in the first steps of autophagy [22]. This is corroborated by the presence of several autophagic proteins at MAMs during starvation, a known stimulus for autophagy. Moreover, the microtubule associated protein light chain 3 (LC3) needs to be conjugated with PE, probably derived from mitochondria, to promote autophagosome elongation and cargo sequestration [221].

### 4.2. MAMs Dysfunction as The Cause of Optic Neuropathy and Neurodegenerative Syndromes

**Figure 4:**
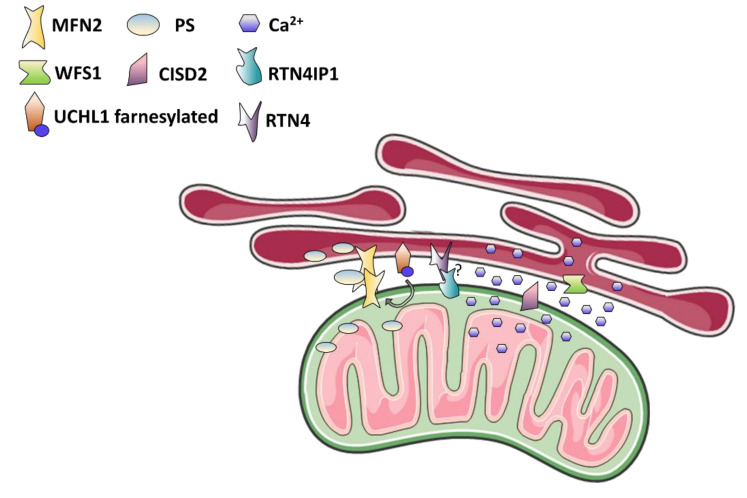
Optic atrophy is related to altered function of several MAMs proteins. A graphical representation of MAMs structure where proteins involved in the degeneration of optic nerve are shown (see also Table 3). PS, phosphatidylserine.

MAMs dysfunction is an emergent pathogenic mechanism in the field of neurodegeneration, which includes also optic neuropathy (Figure 4 and Table 3). As discussed above, *MFN2* mutations cause CMT peripheral neuropathy, with co-occurrence of optic atrophy. Moreover, *MFN2* mutations have deleterious effects on MAMs processes, such as loss of ER-mitochondria contacts or greater inter-organellar distances, activation of UPR, intracellular calcium mishandling, defective PE and PS synthesis, increased production of cholesteryl ester (CE) with accumulation of lipid droplets [137,138]. This latter lipid is in fact produced by the enzyme acyl-CoA:cholesterol acyltransferase 1 (ACAT1), enriched in MAMs, and then stored in the lipid droplets in cytoplasm. Very rarely, *MFN2* homozygous mutations have been reported in patients with multiple lipomatosis and neuropathy [222,223,224], linking MFN2 with lipid metabolism possibly thorough its role in MAMs organization.

Several studies underline this function of MFN2, which was confirmed in lung alveolar type 2 epithelial cells [225], and in conditional Mfn2 knockout adipose-specific mice, demonstrating that Mfn2 mediates mitochondria-lipid droplet interactions and influences lipolytic processes [226]. Furthermore, as discussed above, MFN2 may direct the PS transfer from ER to mitochondria, and alterations of this function leads to hepatic steatosis in mice [214]. Moreover, reduction of MFN2 expression also impairs autophagy and mitophagy, possibly as a consequence of MAMs disruption [227,228], and *MFN2* mutations have been reported to enhance mitophagy in motor neurons [136]. Lipid alterations, autophagy/mitophagy dysfunctions and mtDNA depletion are also related to the lysocardiolipin acyltransferase 1 (ALCAT1), enriched in MAMs, which controls cardiolipin remodeling and MFN2 expression [229,230,231].

Ca^2+^ mishandling and MAMs alterations may be involved in the pathogenic mechanism of two other genes causing complex syndromes with optic atrophy, i.e., *Ubiquitin C-Terminal Hydrolase L1* (*UCHL1*) and *RTN4IP*. Homozygous or compound heterozygous mutations in the *UCHL1* gene, encoding for a neuronal specific deubiquitinating enzyme, have been associated to early-onset spastic paraplegia and optic atrophy, with additional variable features such as peripheral neuropathy, cerebellar ataxia, myokymia, and cognitive impairment [232,233], a phenotype resembling that of Uchl1 knockout mice [234,235]. However, in the most recent KO mouse model optic atrophy was absent, as examined at four months of age [235]. The molecular basis of the neurodegeneration induced by UCHL1 dysfunction has not been investigated yet in patients-derived cells. However, a recent study described a close relationship between UCHL1 and MFN2, demonstrating that loss of UCHL1 impairs mitochondrial morphology, alters the mitochondrial network, oxygen consumption, and, in parallel, reduces ER-mitochondria contact sites and mitochondrial Ca^2+^ uptake [236]. Interestingly, UCHL1, when farnesylated, is associated to ER membranes and, concordantly, it has been detected in MAMs fractions of mouse brain tissues [237,238]. In addition, UCHL1 deficiency and accumulation of polyubiquitinated proteins have been reported in β-cells from type 2 diabetes affected patients, thus connecting again MAMs dysfunction to diabetes [239].

Mutations in the *RTN4IP1* gene are associated with either recessive isolated optic atrophy or severe early-onset encephalopathy [57,58]. RTN4IP1 is a mitochondrial protein with still unknown function. However, it interacts with RTN4 (also known as NOGO), an ER protein enriched in MAMs [57,237], which regulates dendrites formation and extension in the developing central nervous system [240]. Smaller optic disc size in patients carrying *RTN4IP1* mutation and the concordant phenotype of small eyes lacking RGCs in knockdown zebrafish are both indicative of an abnormal development of the optic nerve, possibly due to loss of interaction with RTN4 [57]. Enzymatic activity of complex I and IV was found defective in RTN4IP1 patients-derived fibroblasts, as well as complex I assembly. Mitochondrial network fragmentation has been reported in some but not in all fibroblast cell lines studied [57,58], probably as a secondary effect of mitochondrial dysfunction. Last, RTN4IP1 mutant fibroblasts showed increased sensitivity to UV light-induced damage, suggesting that also the continuous exposure of RGCs to visible light may be deleterious in the presence of defective RTN4IP1 [57].

### 4.3. Wolfram Syndrome: The MAMs Perspective

Another example of optic atrophy related to MAMs dysfunction is Wolfram syndrome (WS1), an autosomal recessive multi-systemic disease characterized by diabetes insipidus, diabetes mellitus, optic atrophy, deafness, and a wide range of additional neurological features, such as cerebellar ataxia, peripheral neuropathy psychiatric disorder, and epilepsy [241]. The genetic cause of WS is the presence of biallelic mutations in the *Wolfram syndrome 1* (*WFS1*) gene, encoding for Wolframin, a protein located in the ER membranes and involved in calcium homeostasis and in the UPR [242,243,244,245,246]. Heterozygous mutations in *WFS1* have also been associated with non-syndromic diseases characterized by isolated hearing loss, diabetes or congenital cataract, and with Wolfram-like syndrome, characterized by progressive hearing impairment, diabetes mellitus, and optic atrophy [247]. Moreover, cases of isolated optic atrophy have been reported as due to biallelic mutations in this gene [248]. A partial overlapping disease, Wolfram syndrome type 2 (WS2), is caused by recessive mutations in the *CDGSH Iron Sulfur Domain 2* (*CISD2*) gene, encoding for a MAM localized protein (also known as Miner1 or ERIS) that, similarly to WFS1, regulates the UPR and calcium homeostasis [249,250,251]. From a clinical perspective, WS2 differs from WS1 for the lack of diabetes insipidus and psychiatric features, and the presence of platelet aggregation resulting in upper gastrointestinal ulceration and bleeding [250,252]. However, a case of homozygous *CISD2* mutation associated with “classical” WS1 has been reported [253]. Interestingly, a proteomic study evidenced the presence of Wfs1 in the MAMs fraction isolated from mouse brain tissues [237], and we recently confirmed that WFS1 is enriched in the MAMs also in human cell lines [19]. These findings suggest that Wolfram syndrome, and associated optic atrophy, may be due to alterations in MAMs-related processes, as in CMT2A. According to this consideration, WS1 fibroblasts show reduced mitochondrial Ca^2+^ uptake driven by altered Ca^2+^ release from ER and altered ER-mitochondria contacts, whereas mitochondrial dynamics and morphology are not affected [18,19]. The increase in non-oxidative glycolysis, documented by high lactate levels in plasma of WS1 patients, in absence of defective mitochondrial respiration, and enhanced extracellular acidification rate (ECAR) in fibroblasts, may be related to a reduced activity of PDH, as regulated by mitochondrial Ca^2+^, and to diabetes [19]. The presence of a mitochondrial bioenergetics dysfunction is still unclear, since controversial results have been reported [18,19]. However, it is possible that, in the specific cell types affected in WS (neurons and pancreatic β-cells), the mitochondrial OXPHOS may be affected due to calcium mishandling. In fact, in neuronal cells from a Wfs1-knockout mouse altered mitochondrial trafficking, inhibition of mitochondrial fusion, and increased mitophagy have been described [254]. Similarly, altered calcium homeostasis due to loss of WFS1 causes cell death of pancreatic β-cells or in patient-derived neuronal progenitor cells through hyper-activation of calpain 2, a calcium dependent protease [255,256]. Furthermore, WFS1 promotes insulin biosynthesis and negatively regulates ER stress inhibiting DNA damage inducible transcript 3 (also known as CHOP)-mediated apoptosis in β-cells, ensuring the physiological function of these cells [257]. Consistent with MAMs dysfunction, ER stress have been documented in the retina from Wfs1-knockout mouse, showing increasing levels of UPR markers (BiP/GRP78, protein disulfide isomerase, and inositol-requiring enzyme 1 alpha) [258], and it is possibly caused by ER permeabilization [259].

WFS1 and CISD2 share similar functions and their genetic alterations cause similar diseases. However, these two proteins probably do not work in synergy, since they are not interactors, and are structurally different, being WFS1 a glycoprotein and CISD2 a redox-active 2Fe2S cluster protein [249,251]. ER stress and activated UPR have been observed in *Cisd2*- knockout mice and MEFs, as well as mitochondrial ultrastructural changes such as increased cristae density [251], also in the optic nerve [260]. Loss of Cisd2 induces a dramatic alteration of intracellular Ca^2+^ consisting in depletion of ER Ca^2+^ storage that, as opposed to WFS1 dysfunction, leads to Ca^2+^ overload in mitochondria. Accordingly, the oxygen consumption rate is increased, possibly secondary to the activation of PDH by Ca^2+^, which boosts the conversion of pyruvate into acetyl-CoA and the production of OXPHOS substrates through the Krebs cycle [251]. Enhanced reactive oxygen and nitrogen species have also been reported in these models [251], confirming previous indications on CISD2 as a master regulator of life-span, possibly through its anti-oxidant activity [260]. Fibroblasts carrying a *CISD2* missense mutation not affecting mRNA or protein expression confirmed the enhanced Ca^2+^ flux from ER to mitochondria, in addition to increase ER-mitochondria contact sites and hyper-fusion of mitochondrial network. No ER stress was found, although the ER lumen was swollen. Moreover, patient-derived fibroblasts showed defective complex I and II activities and reduced ATP levels when grown in glucose-free galactose containing medium, a well-known stress condition that highlights mitochondrial bioenergetics defects [253]. Similarly to WFS1, CISD2 dysfunction induce cell death through calpain 2 activation, as demonstrated in knockdown neuronal and β-cells [256]. Lastly, myelin degeneration has been described in the axons of central (optic) and peripheral (sciatic) nerves, which seems to be mediated by increased autophagy [260]. Considering that abnormal white matter myelination has been described in a young WS1 patient and that ER stress and UPR are implicated in some inherited myelin disorders (for example Vanishing White Matter disease), Wolfram syndrome has also been proposed to be a neurodevelopmental disorder characterized by ER stress-mediated impairment of myelination [261,262].

## 5. Other Emerging Mechanisms: From Oxidative Phosphorylation to Lipid Metabolism

**Figure 5:**
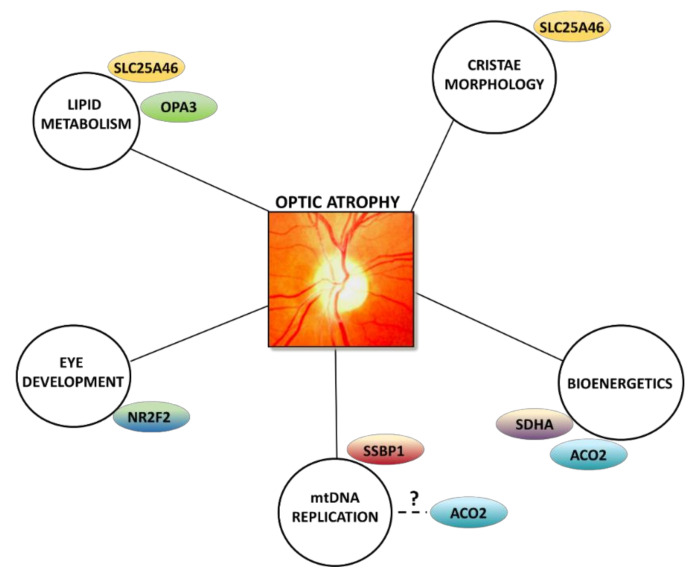
Additional pathways involved in optic atrophy. Schematic representation of different pathways converging on optic nerve degeneration, involving several genes associated with optic atrophy. A pale optic nerve head is shown at the center of the figure.

Additional genes have been implicated in the pathogenesis of optic atrophy, some of them converging on the same, often intercorrelated, pathways, such as mitochondrial morphology and cristae architecture, lipid metabolism, oxidative phosphorylation, and mtDNA maintenance (Figure 5).

Optic Atrophy 3 (*OPA3*) and Solute Carrier Family 25 Member 46 (SLC25A46) are associated with recessive inherited complex neurodegenerative disorders that include optic atrophy as a key feature (Costeff syndrome and CMT with cerebellar atrophy, respectively) [263,264,265]. Moreover, heterozygous mutations in *OPA3* are responsible for DOA, isolated or presenting with cataract and/or hearing loss, and characterized by intra- and interfamilial variability in the phenotypic expression [9,266,267]. Recently, a case of DOA with cataract and axonal neuropathy has also been reported [268]. Additionally, the phenotypic spectrum of SLC25A46 expanded, including the severe infantile Leigh-like encephalopathy [265], but also severe congenital ponto-cerebellar hypoplasia [269,270,271] and more recently optic atrophy and Parkinsonism [272], this latter case resembling some specific OPA1 mutations [73]. Both OPA3 and SLC25A46 proteins are located in mitochondria and have initially been described as mitochondrial fission regulators, since knockdown experiments or studies on patients-derived fibroblasts showed hyperfusion of mitochondrial network [264,265,273,274,275]. However, more recent findings suggest that both OPA3 and SLC25A46 may be involved in lipid homeostasis, and SLC25A46 controls mitochondrial cristae architecture through regulation of the mitochondrial contact site and cristae organizing system (MICOS) complex [265,276,277]. While the role of OPA3 in mitochondrial fission remains unclear, it has been recently demonstrated that SLC25A46 modulates oligomerization and stability of MFN1 and MFN2, resulting in increased mitochondrial fusion upon its loss [278]. OXPHOS dysfunctions have been described in both OPA3 and SLC25A46 patients’ cells [82,264,265].

Alterations in ceramides and sphingomyelins, implicated in cell survival and biological membrane functioning, underlie the pathogenic mechanism of the *Elongation Of Very Long Chain Fatty Acids 1* (*ELOVL1*) gene heterozygous mutations, leading to ichthyosis, hypomyelination, spastic paraplegia, deafness, and optic atrophy [279,280].

Depletion of mtDNA, and consequent OXPHOS dysfunction, due to SSBP1 dominant, but also recessive, mutations are the cause of optic atrophy with retinal abnormalities [12,13,14], in some cases complicated by deafness and renal disease leading to transplant [13]. Indeed, SSBP1 is essential for the correct replication of mtDNA, binding and stabilizing to the single strand of the displacement loop during mtDNA synthesis [281].

Complex II (succinic dehydrogenase, SDH) deficiency due to heterozygous mutations in the *SDHA* gene leads to bilateral optic atrophy, cerebellar atrophy, polyneuropathy, psychiatric involvement, and cardiomyopathy [282]. More recently, an additional case of childhood onset bilateral optic atrophy and cognitive impairment caused by a heterozygous mutation in SDHA has been described [283]. It should be noted that SDH is an enzyme acting both in the mitochondrial respiratory chain and in the Krebs cycle, coupling the oxidation of succinate to fumarate with the reduction of ubiquinone to ubiquinol.

Interestingly, Aconitase 2 (ACO2), another enzyme of the Krebs cycle that converts citrate into isocitrate, is responsible for both recessive and dominant optic neuropathy, isolated or occurring in syndromic forms with encephalopathy and cerebellar atrophy [284,285]. Interestingly, mtDNA depletion has been observed in patients’ fibroblasts carrying a heterozygous *ACO2* mutation leading to haploinsufficiency [285], congruent with another case of recessive neuromuscular disease due to compound heterozygous mutations in *ACO2* [286].

Last, NR2F2, an orphan nuclear receptor ligand-activated transcription factor, is associated with the Bosch-Boonstra-Schaaf optic atrophy syndrome (BBSOSA), characterized by delayed development, moderate intellectual disability, and optic atrophy [287]. Mouse Nr2f2, also known as COUP-TFII, regulates eye development controlling the expression of several regulatory genes essential for early optic vesicle development [288]. Recently, it has been demonstrated that NR2F2 induces oxidative stress and represses several genes required for the maintenance of mitochondrial function and is elevated in dopaminergic neurons of Parkinson disease patients [289].

## 6. Concluding Remarks and Future Directions

**Figure 6:**
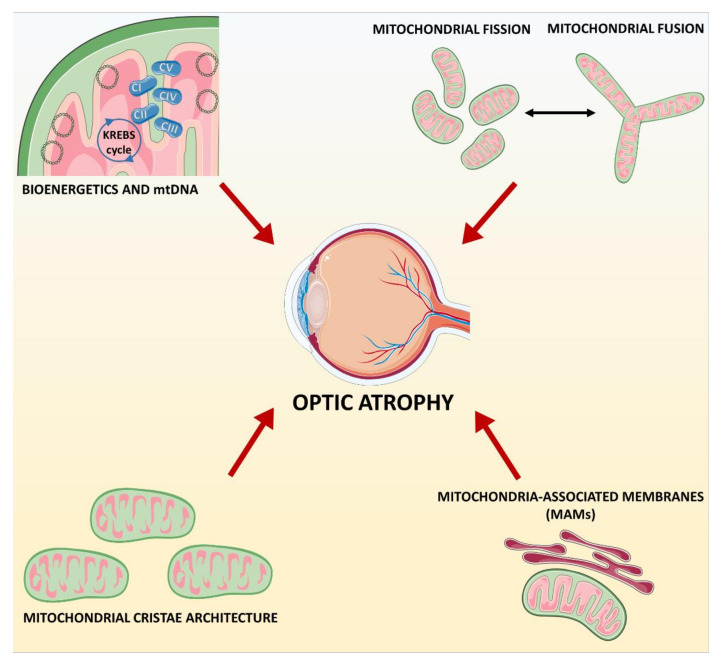
Molecular pathways behind inherited optic atrophy. Bioenergetics, mtDNA maintenance, mitochondrial dynamics, mitochondrial cristae morphology and MAMs are the principal interconnected pathways implicated in the degeneration of optic nerve.

Embracing a holistic view, the key pathological mechanisms underlying the common phenotypic outcome of optic atrophy here delineated, namely dysfunction of complex I, mitochondrial dynamics, and the inter-organelles dialog between ER and mitochondria, are, in turn, all interconnected, in fact also implicating mtDNA maintenance and OXPHOS efficiency, ROS production, lipid biosynthesis and membrane maintenance, and calcium handling (Figure 6). The gap of knowledge that needs to be further and rapidly filled, to truly impact these mechanisms by therapies, is how these pathways and molecular mechanisms translate into neuronal degeneration. It has been emphasized elsewhere the next complexity of applying these mechanisms to the specific cell types involved in the final outcome of neuronal degeneration [22]. These may include glial cells, such as oligodendrocytes providing the myelin wrapping to neuronal axons, but also the vascular tissue or the immune system, which may be implicated in the metabolic regulation and inflammatory responses.

Ultimately, the neuronal metabolism and architecture, which differs in neuronal subtypes, in particular considering the key target for optic neuropathies represented by RGCs, needs to be investigated by directly perturbing the pathologic mechanisms we have reviewed here. This is the ultimate basis to elaborate therapeutic strategies, and to reach this objective the future use of patient-derived reprogramed stem cells and differentiated cells or organoids is one of the innovative tools in addition to and complementing the traditional animal modeling of human diseases [290]. In the context of neuronal cells, key physiological activities, such as axonal transport, maintenance of the synaptic terminals, strict interaction with oligodendrocytes for myelin maintenance, just to name the most relevant, are all areas of research interest [1,2,3,4,23]. Extremely powerful approaches are nowadays available to reach these objectives, such as single cell multiomic analysis, as these may be dissected from organoids and/or human derived tissues when available [291], in conjunction with massive screening for compensatory mechanisms or for identifying effective drugs by CRISPR/Cas9-based technologies [292].

If we look at the pathogenic mechanisms here reviewed, we may already envisage some major areas for therapeutic strategies. As far it concerns strictly complex I, the recent elucidation of the structure and function of this multi-subunit very large enzyme opens the way to tailor drug approaches to the specific defects generated by the different mutations. Downstream consequences may be more relevant on defective bioenergetics or on ROS productions, potentially allowing for more specific interventions, such as metabolic rewiring [103]. Switching on mitochondrial dynamics, besides the possibility to modulate fusion or fission, this is profoundly imbricated with the homeostatic control of mitochondrial biogenesis, as well as with the removal of dysfunctional mitochondria by targeted autophagy [293]. As we emphasized in the previous sections, the inter-organellar communication through the MAM is also directly interconnected with mitochondrial dynamics, and offers another field of therapeutic intervention by modulating the calcium homeostasis. On top of all these possible approaches sits the offer to correct the genetic defects directly by gene therapy or gene editing, which provides a completely different level of therapeutic intervention [294]. The deep understanding of RGCs’ specific developmental programs, in addition to their metabolic and physiological properties, may overcome the challenge of optic nerve regeneration. This ultimate goal will provide a cure to those patients who already suffered the neurodegeneration [295].

## Figures and Tables

**Table 1:** Main genes associated with Complex I dysfunction leading to optic atrophy.

Gene	Genome	Disease	Inheritance	Protein Function
*MT-ND1*	mitochondrial	LHON	MI	CI subunit
*MT-ND4*	mitochondrial	LHON	MI	CI subunit
*MT-ND6*	mitochondrial	LHON	MI	CI subunit
*DNAJC30*	nuclear	LHON	AR	CI turnover
*TMEM126A*	nuclear	OA and auditory neuropathy	AR	CI assembly
*RTN4IP1*	nuclear	OA with or without ataxia, ID, and epilepsy	AR	CI assembly

LHON, Leber Hereditary Optic Neuropathy; MI, maternal inheritance; CI, complex I; AR, autosomal recessive; OA, optic atrophy, ID, intellectual disability.

**Table 2:** Main nuclear genes associated with mitochondrial dynamics dysfunction leading to optic atrophy.

Gene	Effect on Mitochondrial Network	Disease	Inheritance	Protein Function
*OPA1*	Fragmented	DOA DOA-plus	AD	IMM fusion
*YME1L*	Fragmented	Mitochondrial encephalopathy with OA	AR	OPA1 processing
*AFG3L2*	Fragmented	DOA with or without additional neurological features, CPEO	AD	OMA1 regulation
*SPG7*	Normal or hyperfused	DOA Spastic paraparesis and OA, CPEO	AD AR	OMA1 regulation
*MFN2*	Fragmented or hyperfused	CMT with or without OA HMSN Lipomatosis and neuropathy	AD/AR AD AR	OMM fusion; MAMs formation; PS transfer
*DRP1*	Hyperfused	Mitochondrial encephalopathy with OA DOA	AD/AR AD	OMM fission
*MFF*	Hyperfused	Mitochondrial encephalopathy with OA	AR	DRP1 adaptor
*MIEF1*	Fragmented	DOA	AD	DRP1 adaptor
*GDAP1*	Fragmented or hyperfused	CMT with or without OA	AD/AR	DRP1 and FIS1 regulation?

DOA, Dominant Optic Atrophy; AD, autosomal dominant; IMM, inner mitochondrial membrane; OA, Optic Atrophy; AR, autosomal recessive; CPEO, Chronic Progressive External Ophthalmoplegia; CMT, Charcot-Marie-Tooth neuropathy; HMSN, Hereditary motor and sensory neuropathy; MAMs, mitochondria-associated membranes; PS, phosphatidylserine.

**Table 3:** Main nuclear genes associated with mitochondria-associated membranes dysfunction leading to optic atrophy.

Gene	Effect on MAMs Function	Disease	Inheritance	Protein Function
*MFN2*	Reduced ER-mitochondria contacts; UPR activation; Ca^2+^ and lipids mishandling; altered autophagy	CMT with or without OA HMSN Lipomatosis and neuropathy	AD/AR AD AR	OMM fusion; MAMs formation; PS transfer
*UCHL1*	Reduced ER-mitochondria contacts; Ca^2+^ mishandling ^1^	Spastic paraplegia with OA	AR	Protein deubiquitination
*RTN4IP1*	Unknown	OA with or without ataxia, ID, and epilepsy	AR	CI assembly; RTN4 interactor
*WFS1*	Reduced ER-mitochondria contacts; Ca^2+^ mishandling	WS1 Isolated cataract, or diabetes or deafness Isolated OA WS-like	AR AD AR AD	Ca^2+^ homeostasis; UPR and ER stress regulation; insulin biosynthesis
*CISD2*	Increased ER-mitochondria contact; mitochondrial Ca^2+^ overload; enhanced ROS	WS2	AR	Ca^2+^ homeostasis; anti-oxidant activity

^1^ Effects of UCHL1 KD in human cells. UPR, unfolded protein response; CMT, Charcot-Marie-Tooth; OA, optic atrophy; HMSN, Hereditary motor and sensory neuropathy; AD, autosomal dominant; AR, autosomal recessive; OMM, outer mitochondrial membrane; MAMs, mitochondria-associated membranes; PS, phosphatidylserine; ID, intellectual disability; WS1, Wolfram syndrome type 1; WS2, Wolfram syndrome type 2; ROS, reactive oxygen species.

## Data Availability

Not applicable.
